# Is Multiple Sclerosis an Extra-Intestinal Manifestation of Inflammatory Bowel Disease? Food for Thought

**DOI:** 10.7759/cureus.9485

**Published:** 2020-07-30

**Authors:** Karolina N Dziadkowiec, Peter Stawinski, Dhruvil Radadiya, Baher Al Abbasi, Shaun Isaac

**Affiliations:** 1 Internal Medicine, University of Miami, John F. Kennedy Regional Campus, Atlantis, USA; 2 Internal Medicine, East Tennessee State University, Johnson City, USA; 3 Internal Medicine, University of Miami, John F. Kennedy Medical Center, Atlantis, USA

**Keywords:** multiple sclerosis, crohn's disease, ibd, ms, colonoscopy, egd

## Abstract

For many years there has been a suggested association between multiple sclerosis (MS) and inflammatory bowel disease (IBD). Aside from their common epidemiological and immunological similarities, there appears to be an association between the incidence of both diseases coexisting. We report a case of a 41-year-old man with chronic diarrhea and weakness, who was found to have concomitant MS and Crohn's Disease. Our report underscores the importance clinicians of maintaining a high degree of suspicion about the potential association of these conditions among these patient populations.

## Introduction

The association between multiple sclerosis (MS) and inflammatory bowel disease (IBD), has been supposed for decades [1]. Emerging literature suggests that this relationship is far more common and is likely underreported [2]. Both diseases appear to share common immunological and epidemiological similarities [3]. The association between MS and IBD has been suggested by the observation of an increased incidence of IBD among MS patients and similarly, an increased incidence of MS among IBD patients [4,5]. Understanding this association has become increasingly important in recent years, as autoimmune diseases become more prevalent.

## Case presentation

A 41-year-old Hispanic man with no significant past medical history presented to the hospital for difficulty walking and diarrhea. Over the last four years, he has had watery, non-bloody diarrhea, with multiple episodes occurring daily. He reported one bloody bowel movement one week prior to presentation. He noted losing a significant amount of weight, despite a good appetite over a three- to six-month period.

**Figure 1:**
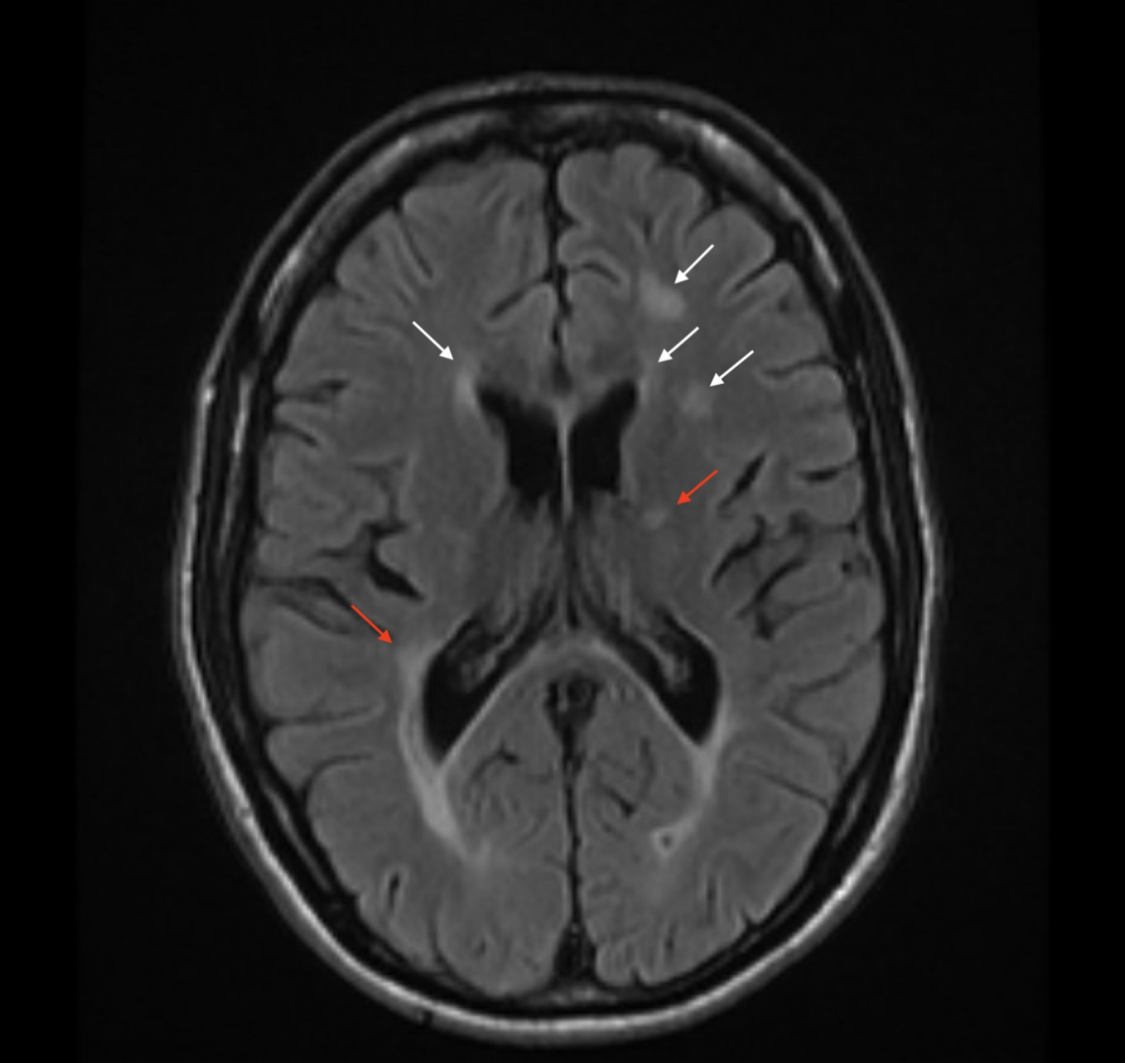
Axial T2 flair magnetic resonance imaging (MRI) of the brain revealing periventricular demyelinating process (white arrows) and white matter inflammatory changes around the perimedullary veins, known as Dawson Fingers (red arrows) consistent with multiple sclerosis

His difficulty walking began approximately two years prior to presentation and had progressively worsened. He described his lower extremities as feeling heavy and numb, and his ability to walk acutely worsened two months prior to presentation. Physical examination showed a cachectic patient with temporal wasting. Neurological examination demonstrated bilateral lower extremity weakness involving the hips, knees, and ankles (left side was more affected than the right side), hyperreflexia of the bilateral patellar jerk and bilateral extensor plantar response, decreased vibration and pinprick sensations bilaterally - up to 8 centimeters above the ankles. All remaining neurological examinations were intact. Abdominal examination was benign. Initial workup revealed a C-reactive protein (CRP) of 1.8. MRI of brain and spine demonstrated an advanced demyelinating process in the brain and in the mid- and lower-cervical spinal levels of the spinal cord - suspicious for MS (Figure 1).

Cerebrospinal fluid (CSF) cytology demonstrated red-blood cells (RBC) of 3, glucose of 70, white blood cells (WBCs) of 0 and a protein level of 53 with immunoglobulin G (IgG) oligoclonal bands (IgG index of 1.5; normal range is 0-0.7). Based on the McDonald criteria, he was diagnosed with primary progressive MS. Subsequently, colonoscopy and esophagogastroduodenoscopy (EGD) were pursued and biopsies obtained during the procedure confirmed the diagnosis of ileocolonic Crohn’s disease. The patient was subsequently initiated on high dose intravenous pulse steroids for five days and then transitioned to oral therapy. He had a very timely and remarkable improvement of his weakness and diarrhea. The patient was discharged and scheduled to follow up in the outpatient setting with gastroenterology and neurology.

## Discussion

IBDs, including ulcerative colitis (UC) and Crohn’s disease (CD), are relapsing-remitting, idiopathic and chronically debilitating conditions. Specifically, CD has the potential to transmurally affect the entire gastrointestinal tract and is associated with various extra-intestinal manifestations; in contrast, UC typically affects the large intestine [6,7]. The relationship between MS and IBD was first suggested by Rang et al. in 1982; the prevalence of MS was approximately three-fold higher in patients who had undergone a colectomy for the treatment of IBD [8]. Similarly, a retrospective study also detected an increased incidence of demyelinating disease among patients with IBD, and especially among patients with UC [9]. 

The estimated prevalence of MS in the general population is approximately 0.1%; conversely, in patients suffering from IBD the prevalence of MS has been reported up to 0.5%. These data suggest a 1.5 to 5-fold increase in the risk of the development of MS in patients with IBD [10,11]. The pathogenesis of neurogenic disorders associated with IBD has not been fully established and is possibly related to a multitude of environmental, genetic and immunological causes [12]. The failure of the innate immune system and gastrointestinal mucosal integrity appears to play a large role in the development of IBD. Some studies suggest that certain T-cell lines with abnormal proinflammatory activity of T-helper 17 subsets have been implicated in the pathogenesis of IBD [13]. As a result of this potential association, an MRI of the brain and spinal cord should be obtained, followed by a neurological consultation for further diagnostic workup as soon as a patient with a clinical history of IBD presents with a neurological symptom suggestive of MS or other neurological complaints [14].

## Conclusions

The exact causes of Crohn’s disease and ulcerative colitis continue to be poorly understood. Identification of the association between IBD and demyelinating diseases may help to identify common environmental and genetic factors contributing to the development of these diseases. There may be a significant relationship between MS and IBD; demyelinating disorders appear to be more prevalent in patients with IBD than in the general population. Albeit these diseases have an undetermined etiology, it is suggested that genetic predisposition, disruption of the intestinal microbial flora and the immune system of the gastrointestinal tract appear to play significant roles. Recognition of this association by clinicians may identify patients at greater risk; however, this relationship requires future studies in larger populations to clarify this association.
